# A New Training Assessment Method for Alpine Ski Racing: Estimating Center of Mass Trajectory by Fusing Inertial Sensors With Periodically Available Position Anchor Points

**DOI:** 10.3389/fphys.2018.01203

**Published:** 2018-08-30

**Authors:** Benedikt Fasel, Matthias Gilgien, Jörg Spörri, Kamiar Aminian

**Affiliations:** ^1^Laboratory of Movement Analysis and Measurement, École Polytechnique Fédérale de Lausanne, Lausanne, Switzerland; ^2^Department of Physical Performance, Norwegian School of Sport Sciences, Oslo, Norway; ^3^Center of Alpine Sports Biomechanics, St. Moritz Health and Innovation Foundation, St. Moritz, Switzerland; ^4^Department of Sport Science and Kinesiology, University of Salzburg, Salzburg, Austria; ^5^Department of Orthopaedics, Balgrist University Hospital, University of Zurich, Zurich, Switzerland

**Keywords:** alpine ski racing, giant slalom, center of mass, kinematics, inertial sensors, sensor fusion, validation

## Abstract

In this study we present and validate a method to correct velocity and position drift for inertial sensor-based measurements in the context of alpine ski racing. Magnets were placed at each gate and their position determined using a land surveying method. The time point of gate crossings of the athlete were detected with a magnetometer attached to the athlete’s lower back. A full body inertial sensor setup allowed to track the athlete’s posture, and the magnet positions were used as anchor points to correct position and velocity drift from the integration of the acceleration. Center of mass (CoM) position errors (mean ± standard deviation) were 0.24 m ± 0.09 m and CoM velocity errors were 0.00 m/s ± 0.18 m/s. For extracted turn entrance and exit speeds the 95% limits of agreements (LoAs) were between −0.19 and 0.33 m/s. LoA for the total path length of a turn were between −0.06 and 0.16 m. The proposed setup and processing allowed estimating the CoM kinematics with similar errors than known for differential global navigation satellite systems (GNSS), even though the athlete’s movement was measured with inertial and magnetic sensors only. Moreover, as the gate positions can also be obtained with non-GNSS based land surveying methods, CoM kinematics may be estimated in areas with reduced or no GNSS signal reception, such as in forests or indoors.

## Introduction

In the development of World class athletes’ monitoring, assessment of their training quantity and quality and evaluation of their performance plays a substantial role also in snow sports. Among snow sports, alpine skiing sets high demands to measurement systems to assess training load and performance: the athlete’s speed is high and they cover distances of several hundred meters or kilometers during competition and training in harsh outdoor conditions. Video and body worn sensor-based systems have been proposed to assess performance (Supej et al., 2005; Reid, 2010; Spörri et al., 2012) and training load (Spörri et al., 2015, 2017; Gilgien et al., 2018). To allow teams and athletes to use technology in the training assessment its user friendliness is a key aspect. The use of quantitative video-based analysis is extremely resource consuming and is therefore seldom applied, while the use of body worn sensor technology has increased substantially the last years, due to the efficiency of its application. While quantitative video-based analysis was proven having sufficient accuracy, body worn sensors need still further prove whether their accuracy is sufficient for its applications. To measure human body displacement in alpine ski racing using body worn sensors, differential global navigation satellite systems (GNSS) are recognized to be well suited. They allow obtaining the three dimensional (3D) antenna trajectory at a reasonably high sampling frequency with sub 5-cm accuracy for good GNSS conditions (Gilgien et al., 2014). For applications where overall body posture remains relatively constant, it can be assumed that the 3D center of mass (CoM) kinematics can be approximated by the GNSS antenna kinematics with sufficient precision (Terrier and Schutz, 2005; Townshend et al., 2008; Waldron et al., 2011; Scott et al., 2016). However, when body posture is changing significantly during motion cycles, and when instantaneous CoM kinematics are the variables of interest, an approximation of CoM by the kinematics of a GNSS antenna cannot be considered to be sufficiently valid. Thus, an alternative solution needs to be found to track the athlete’s CoM 3D position relative to the GNSS antenna position.

The determination of the athlete’s absolute 3D CoM position consists of two aspects: (1) the global GNSS antenna position in 3D and (2) the relative position of the CoM with respect to the GNSS antenna position in 3D. To this end, for alpine ski racing, two solutions were proposed: either a modeling approach (Supej et al., 2013; Nemec et al., 2014; Gilgien et al., 2015c) or, more commonly, a combination or fusion of GNSS with inertial sensors (Brodie et al., 2008; Supej, 2010; Fasel et al., 2016a). Generally, both solutions allow the estimation of absolute CoM trajectory with an accuracy and precision of <0.1 m, provided that differential GNSS is used. However, the use of differential GNSS has also two major drawbacks: (1) geodetic differential GNSS hardware and software are very costly and need to be handled by trained personnel and (2) good satellite coverage is needed, indeed signal shading by forest or topography is not unusual in competitive alpine ski racing. Thus, for routine measurements (e.g., during training sessions) this setup might have the disadvantage of being cumbersome and requires personnel trained in geodesy. Therefore, alternatives to measure CoM kinematics should be found.

As already mentioned, inertial sensors can be used to estimate the athlete’s relative 3D CoM kinematics (i.e., relative CoM position with respect to a point on the athlete such as the head). For example, for indoor carpet skiing, an accuracy and precision of 0.03 and 0.01 m was found for the CoM position relative to the lumbar joint center (LJC) (Fasel et al., 2017c). The relative position of the athlete’s head with respect to the LJC could be estimated with an accuracy and precision of 0.13 and 0.02 m, respectively (Fasel et al., 2017c). Considering the above-mentioned drawbacks, finding new solutions to estimate not only the relative but also the absolute CoM position would render the use of differential GNSS obsolete. However, the problem of inertial sensors is that they cannot measure the position directly. Instead, measured acceleration in the sensor frame has to be transformed into a global frame, Earth’s gravity removed, and then integrated twice to finally obtain position. Eventual measurement errors from the first two steps may accumulate during the integration, resulting in large position drifts (i.e., the main limit of such methodology).

Biomechanical movement constraints can help to correct this drift. For example, in gait analysis where inertial sensors are fixed to the feet, drift can be reduced by setting speed to zero at each stance phase (Mariani et al., 2010). However, for activities without motionless periods, e.g., skiing where no stance-swing phases are present, this procedure cannot be applied. When combining inertial sensors and GNSS, 3D speed and position obtained with inertial sensors can be corrected periodically, each time a new GNSS reference sample is available (Grewal et al., 2013). However, such position reference samples (i.e., anchor points) may also come from a difference source, independent from a GNSS. If such anchor points are available at a sufficiently high rate, they could entirely replace the GNSS. If the athlete crosses *a priori* known locations and the corresponding times of crossing can be determined, these locations could be used as anchor points to correct the position drift from the integration of the acceleration. In alpine ski racing the athlete is constrained to follow a pre-defined path marked by gates. Therefore, these gates could be considered as potential anchor points. If the gate locations and the corresponding times of gate crossings are known, position drift could be corrected. Hence, it might be possible to measure an athlete’s CoM trajectory by the sole use of inertial sensors (i.e., without any differential GNSS data being required). Gate locations could be measured using land surveying techniques (Gilgien et al., 2015a,b). Gate crossing times could be obtained by a magnetometer-based method as presented in Fasel et al. (2016c).

Accordingly, the aim of this study was to design and validate a system to estimate absolute 3D CoM kinematics during alpine giant slalom (GS) and downhill (DH) skiing without the use of GNSS (i.e., by the sole use of inertial sensor measurements fused with gate timing and gate position information as anchor points). In order to highlight the system’s relevance for training, skiing performance related parameters derived from the CoM kinematics were tested for sensitivity to change.

## Materials and Methods

### Experimental Setup

**FIGURE 1:**
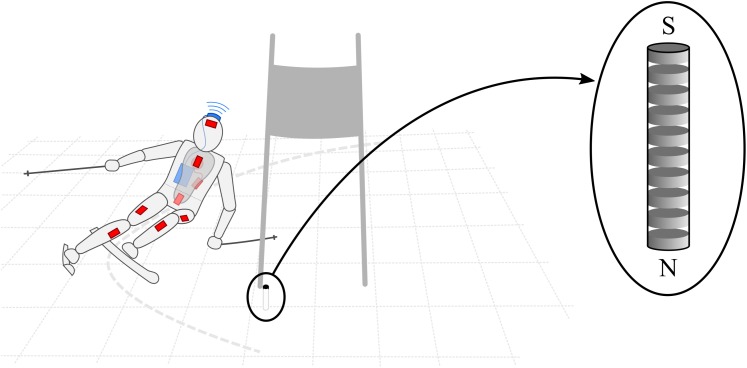
Illustration of the experimental setup during a left turn. The inertial sensors are represented by the red boxes. The reference differential GNSS system is illustrated in blue with the antenna fixed to the helmet and the data logger worn on the back. The back protector contained the low-cost GNSS system with the antenna located approximately between the shoulder blades, as well as the data logger integrated in the inertial sensor fixed to the protector’s left side. The magnets were completely buried into the snow, close to the gate’s pole. A zoomed view of the buried magnet is provided on the right side of the illustration.

Seven inertial sensors (Physilog 4, Gait Up SA, Switzerland) recording acceleration and angular velocity at 500 Hz were fixed to the left and right shanks and thighs, to the sacrum, to the sternum, and to the helmet using medical tape (**Figure 1**). Additionally, the sacrum sensor contained a magnetometer sampling at 125 Hz. Accelerometer offset and sensitivity were corrected according to Ferraris et al. (1995). Gyroscope offset was corrected during a static moment before each run. Magnetometer offset, sensitivity and axis-misalignment were corrected according to Bonnet et al. (2009). A low-cost GNSS receiver (CAM-M8, u-blox, Switzerland) was placed in the athlete’s back protector together with a GNSS antenna (TW2710, Tallysman, Canada) placed approximately at shoulder height. All inertial sensors and the GNSS receiver were wirelessly synchronized. Prior to each run, athletes performed functional calibration movements as described in Fasel et al. (2017d) to align the sensor frames to the anatomical frames of their respective segments. An additional static upright posture with slightly flexed knees and parallel skis was performed at the start and finish. Each gate of a GS course served as an anchor point and was equipped with a magnet. The magnet was constructed by vertically stacking 10 small neodymium magnets (S-20-10-N, Supermagnete, Switzerland) spaced by 5 mm to a 15 cm long stick (**Figure 1**). Magnet position at each gate was obtained using differential GNSS. Thus, each anchor point corresponds to a gate position, which was assumed to be identical to the magnet position.

### Measurement Protocol

Eleven European Cup level athletes performed a total of 17 runs on a typical 30-gates GS course with varying gate distances (21.8–27.8 m). Measurements were performed during four consecutive days. For each day a new course with similar specifications was set. The length of the course from start to finish was 700 m with a vertical drop of 150 m. Every day the position of each gate was geodetically surveyed using GNSS. All athletes gave written informed consent prior to the measurements and the study was approved by the Ethical committee of the École Polytechnique Fédérale de Lausanne (Study Number: HREC 006-2016).

### Simulation of DH Conditions

In DH, gate distances are roughly three times larger compared to GS (Gilgien et al., 2015a,b). Hence, distances between anchor points for the trajectory drift correction are larger and a decreased drift correction performance is expected. DH was simulated by considering only anchor points at every third GS gate for extended Kalman smoothers (EKS) fusion procedure.

### Inertial System Algorithm

#### Data Processing Overview

**FIGURE 2:**
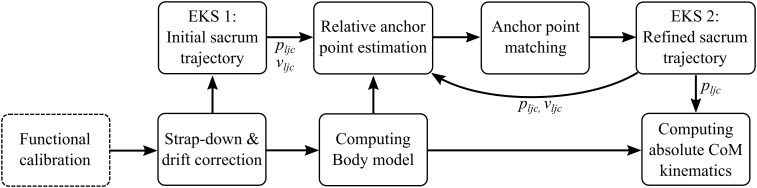
Flow chart of the proposed algorithm for data processing. The outputs of the functional calibration were the rotation matrices that allow an alignment of each inertial sensor with the functional segment frames. The strap-down and drift correction provided the segment orientations in a common global frame. During the EKS 1 the initial sacrum (i.e., lumbar joint center, LJC) position and speed were obtained. Computing of the body model provided the athlete’s joint and CoM positions relative to his LJC. The relative anchor point estimation provided the absolute gate positions in the inertial sensor frame and the relative gate positions at gate crossing with respect to the athlete’s LJC. The anchor point matching computed the transformation between the estimated gate positions expressed in the inertial sensor frame and the global Earth reference frame and matches all estimated gate positions to the surveyed gate positions. The output of EKS 2 was a refined sacrum trajectory and speed. The output of the absolute CoM kinematics computation step was the final estimate of the position and speed of the CoM.

After functional calibration, segment orientation was found with strap-down integration and joint orientation drift correction as described previously (Fasel et al., 2017d, 2018). To fuse the anchor points with acceleration data from the inertial sensor at the sacrum, two separate EKS (Hartikainen et al., 2011) were used. The first smoother was used to obtain an initial 3D sacrum trajectory based on the inertial data only (**Figure 2**). Since the sacrum sensor would not pass the anchor points (i.e., gates) with zero distance, the relative position offsets between each anchor point and sacrum sensor position at gate crossing had to be estimated (relative anchor point estimation). Next, the anchor points estimated with the inertial data were matched to the surveyed anchor points. Then, the second smoother fused the anchor points with the inertial data for obtaining a refined sacrum trajectory. Relative anchor points were re-estimated and matched again to the surveyed anchor points and the EKS was run a second time. Finally, the athlete’s absolute CoM kinematics was determined by combining the sacrum’s refined trajectory with the relative CoM position (**Figure 2**).

#### Extended Kalman Smoother 1: Initial Sacrum Trajectory

The sacrum sensor’s acceleration was expressed in the global frame (*X*-axis: forward with respect to the athlete’s still posture at start; *Y*-axis: vertical, along Earth’s gravity; *Z*-axis: cross-product between *X*- and *Y*-axis; and origin: sacrum position at start) and gravity was removed. To estimate the sacrum trajectory *p*_sacr_(t) an EKS with 15 states (3D position, 3D speed, 3D acceleration, 3D position offset, and 3 Euler angles representing residual orientation drift) integrated the gravity-corrected acceleration twice. A zero-velocity constraint during the static moments at start and finish was used to reduce the position drift. A constant-acceleration model was used for the state transitions.

#### Body Model and Relative Anchor Point Estimation

The athlete’s body model was obtained as described previously (Fasel et al., 2017c) with the LJC as the origin of the athlete’s local coordinate system. Lower limb joint positions and athlete’s CoM were estimated relative to the LJC. As for the sacrum’s initial trajectory, azimuth was set to 0° at the static posture at start. Gate crossings were detected based on the peaks in the recorded magnetic field intensity at the sacrum sensor (Fasel et al., 2016c). For all further processing, it was assumed that the sacrum sensor position was at the same position as LJC [i.e., *p*_sacr_(*t*) = *p*_ljc_(*t*) and in consequence *v*_sacr_(*t*) = *v*_ljc_(*t*)].

**FIGURE 3:**
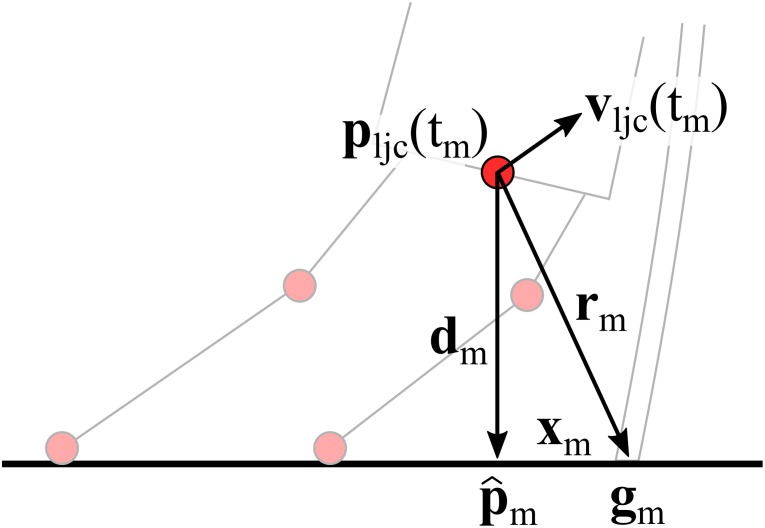
Athlete posture relative to the gate seen from the back at gate crossing illustrated for a right turn. To simplify computation, it was assumed that the inertial sensor fixed to the sacrum would measure the acceleration, angular velocity, and magnetic field at the position of the LJC.

Suppose the skiing course consisted of *N* gates equipped with magnets and *M* gate crossings were detected [where *M* may be different from *N* due to missed gates (i.e., too wide distance from the magnet) or wrong detections due to signal noise]. The *N* gates’ magnet positions (i.e., anchor points) are denoted by {*g*_n_}, *n* ∈ [1; *N*]. The *M* “hypothetical” anchor points are denoted by {*g*_m_}, *m* ∈ [1; *M*]. Suppose further that the LJC trajectory is denoted by *p*_ljc_(*t*) with *t* being time, and that LJC speed is denoted by *v*_ljc_(*t*). For a given gate crossing *m*, detected at time *t*_m_, the vector *r*_m_ is relying *p*_ljc_(*t*_m_) to *g*_m_ and ${\hat{p}}_{\text{m}}$ is the projection of *p*_ljc_(*t*_m_) onto the snow surface *S*_m_ at gate *m*. *x*_m_ is the vector connecting ${\hat{p}}_{\text{m}}$ to *g*_m_ and is assumed to lie on *S*_m_ and perpendicular to *v*_ljc_(*t*_m_) (**Figure 3**). |*r*_m_|| can be estimated based on the magnetic field intensity at gate crossing, ||*B*(*t*_m_)||. For a magnetic point source, its magnetic field intensity ||*B*|| decays exponentially to the third power of the distance ||*r*|| (Furlani, 2001). For the magnets used in this study, based on in-lab measurements with constant ambient magnetic field, the relation of ||*B*|| to ||*r*|| was approximated with Equation 1.

(1) $$\left\|r\right\|=\{\begin{array}{ll} -0.4*\left\|B\right\|+1.0 & \mathrm{if}\;\left\|B\right\|<1.62\\ -0.062*\left\|B\right\|+0.452 & \mathrm{else} \end{array}$$

where the magnetometer was calibrated such that ||*B*|| = 1 for ||*r*|| >> 0.

However, *B*(*t*_m_) did not allow a precise estimate of the xyz-components of *r*_m_. Instead it was computed following Equations 2–4 for right turns and Equations 2–3 and 5 for left turns, using the trigonometric relations as illustrated in **Figure 3**. A turn was labeled as “right turn” if the sacrum’s angular velocity along the trunk’s longitudinal axis was negative at gate crossing.

(2) $$r_{\text{m}}=d_{\text{m}}+x_{\text{m}}$$

(3) $$d_{\text{m}}={\hat{p}}_{m}-p_{\text{lcj}}(t_{\text{m}})$$

(4) $$x_{\text{m}}=\sqrt{{\left\|r_{\text{m}}\right\|}^{2}-{\left\|d_{\text{m}}\right\|}^{2}}*\frac{d_{\text{m}}\times v_{\text{m}}}{\left\|d_{\text{m}}\times v_{\text{m}}\right\|}$$

(5) $$x_{\text{m}}=\sqrt{{\left\|r_{\text{m}}\right\|}^{2}-{\left\|d_{\text{m}}\right\|}^{2}}*\frac{v_{\text{m}}\times d_{\text{m}}}{\left\|v_{\text{m}}\times d_{\text{m}}\right\|}$$

To estimate *S*_m_, first, snow contact points of the left and right feet were obtained by combining *p*_ljc_(*t*) with the athlete’s body model. It was supposed that the contact point of each leg was located 0.15 m distally from its ankle joint center, along the shank’s longitudinal axis. Second, the average ski line *l*(*t*) was computed by averaging between the left and right contact points. Finally, *S*_m_ was obtained by fitting a plane to *l*(*t*), *t* ∈ [*t*_m_ − 0.4*sec*; *t*_m_ + 0.4 *sec*]. Thus, ${\hat{p}}_{\text{m}}$ could be computed according to Equation 6.

(6) $${\hat{p}}_{\text{m}}=p_{\text{ljc}}(t_{\text{m}})+\left(\left({\bar{l}}_{\text{m}}-p_{\text{ljc}}(t_{\text{m}})\right)\cdot n_{\text{m}}\right)*n_{\text{m}}$$

where ${\bar{l}}_{\text{m}}$ is a random point on *S*_m_ (e.g., average of *l*(*t*), *t* ∈ [*t*_m_ − 0.4*sec*; *t*_m_ + 0.4 *sec*]), *n*_m_ the normal vector of *S*_m_, and ⋅ the dot product.

#### Anchor Point Matching

The matching of {*g*_n_}, *n* ∈ [1; *N*] with {*g*_m_}, *m* ∈ [1; *M*] was conducted under the hypothesis that not all N anchor points may have been detected and that additional anchor points may have been wrongly found due to noise in the recorded magnetic field. Since {*g*_m_} are expressed in the inertial sensor’s global frame *J* where both the position and azimuth were initialized to zero during the static posture performed at start, the transformation from *J* to the global Earth reference frame _
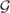
_ had to be found first. Note that the vertical axes of *J* and _
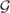
_ were already aligned and that only an azimuth rotation angle α and translation *o* had to be found. To this end, both {*g*_n_} and {*g*_m_} where interpreted as point clouds. The azimuth rotation angle was defined as the angle between the first principal components of {*g*_n_} and {*g*_m_} projected onto the horizontal plane. To find *o*, {*g*_n_} needed to be matched to {*ĝ*_m_}, the azimuth aligned point cloud of {*g*_m_}. To find the best matching solution, a feature vector *f*_n_ and *f*_m_ was constructed for each point in {*g*_n_} and {*ĝ*_m_}, respectively. To construct the features, each anchor point was described relative to its preceding and following anchor point. In addition, each turn was labeled as left/right based on the measured angular velocity and was assigned a turn number (Equations 7–8). To assign turn numbers it was assumed that the first detected anchor point was turn number one and that no two consecutive left or right turns could occur. For each point in {*ĝ*_m_}, the closest matching point *k*_m_ in {*g*_n_} was then found by the minimization of Equation 9. Matchings were removed if two or more points of {*ĝ*_m_} were matched to the same point in {*g*_n_}. *o* was then defined as the median position difference of all matched pairs.

(7) $$\begin{array}{cccc} f_{\text{n}}=[g_{\text{n}+1}-g_{\text{n}}, & g_{\text{n}}-g_{\text{n}-1}, & [l/r], & n{]}^{\text{T}} \end{array}$$

(8) $$\begin{array}{cccc} f_{\text{m}}=[{\hat{g}}_{\text{m}+1}-{\hat{g}}_{\text{m}}, & {\hat{g}}_{\text{m}}-{\hat{g}}_{\text{m}-1}, & [l/r], & m{]}^{\text{T}} \end{array}$$

(9) $$\begin{array}{lll} k_{\text{m}} & = & \arg\min\left\|f_{\text{n}}-f_{\text{m}}\right\|\\ & & n\in [1;N] \end{array}$$

Subsequently, {*g*_m_} was corrected for azimuth and position offset and expressed in frame _
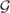
_. Denote these points as {^
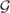
^*g*_m_}. To find the final matching between the estimated anchor points {^
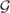
^*g*_m_} and the surveyed anchor points {*g*_n_} the same minimization as described above was used a second time. However, since offset was corrected, feature vectors finally consisted of the absolute position, the left/right turn, and the turn number (Equations 10, 11).

(10) $$\begin{array}{ccc} f_{\text{n}}=[g_{\text{n}}, & [l/r], & n{]}^{\text{T}} \end{array}$$

(11) $$\begin{array}{ccc} f_{\text{m}}={[}^{𝒢}g_{\text{m}}, & [l/r], & m{]}^{\text{T}} \end{array}$$

#### Extended Kalman Smoother 2: Refined Sacrum Trajectory

As expected, the sacrum trajectory *p*_ljc_(*t*) which was solely obtained by integration of the sacrum acceleration and by zerovelocity drift correction was not very accurate and position drifts of up to 20 m were observed. Therefore, an accurate estimation of {*g*_m_} could not be guaranteed and not all matching pairs *k*_m_ were identifiable. Thus, after a first passage through the EKS, the estimation of {*g*_m_} and the anchor point matching were performed a second time. But this time it was based on the updated sacrum trajectory. Finally, the EKS was run a second time to obtain an improved estimation of the sacrum’s trajectory. To account for the improved accuracy of {*g*_m_} the position accuracy of {*g*_m_} in the EKS was reduced from 1 m for the first iteration to 0.1 m for the second iteration.

#### Absolute CoM Kinematics Estimation

Finally, the absolute CoM trajectory *p*_CoM, inertial_(*t*) was obtained by adding the relative CoM position obtained from the body model to the refined sacrum trajectory (Fasel et al., 2016a, 2017c). The athlete’s CoM speed *v*_CoM, inertial_(*t*) was obtained by the three-point derivation of *p*_CoM, inertial_(*t*). Both *p*_CoM, inertial_(*t*) and *v*_CoM, inertial_(*t*) were low-pass filtered with a 2^nd^ order Butterworth filter with cut-off frequency of 5 Hz.

### GNSS Reference System

The reference system consisted of a differential geodetic GNSS with the GNSS antenna (G5Ant-2AMNS1, Antcom, Canada) fixed to the athlete’s helmet. The receiver (Alpha-G3T, Javad, United States) was placed in a backpack and logged GPS and GLONASS signals using the L1 and L2 frequencies. A reference base station (receiver: Alpha-G3T, Javad, United States; antenna: GrAnt, Javad, United States) was placed at the end of the ski course. 3D antenna positions were sampled at 50 Hz and obtained in post processing as described in (Gilgien et al., 2013, 2015c). Ambiguities were fixed for the entire run for all runs. Synchronization with the inertial sensor-based system was performed with the GPS timestamp. To obtain antenna trajectory at 500 Hz the antenna position samples were fused with the head’s inertial sensor data using an EKS with twelve states (3D position, 3D speed, 3D acceleration, and 3D acceleration offsets). This trajectory was then combined with the athlete’s body model derived from the inertial sensors described and validated in (Fasel et al., 2016a, 2017c) to obtain the reference 3D CoM trajectory *p*_CoM,ref_(*t*). 3D CoM speed *v*_CoM,ref_(*t*) was obtained by three-point derivation of *p*_CoM,ref_(*t*). In the end, both *p*_CoM,ref_(*t*) and *v*_CoM,ref_(*t*) were low-pass filtered with a 2nd order Butterworth filter with cut-off frequency of 5 Hz.

### Validation

#### CoM Kinematics

**FIGURE 4:**
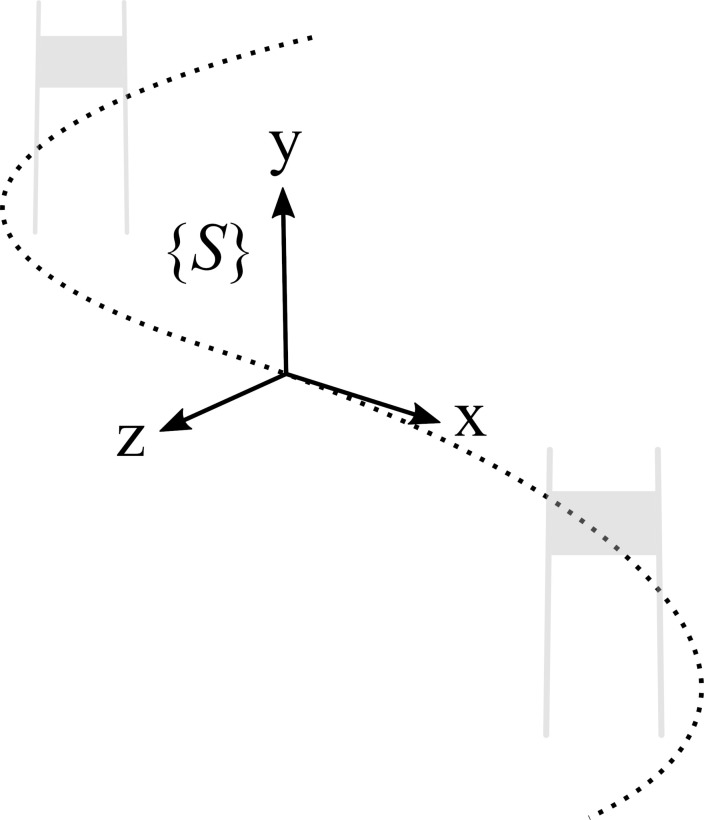
Definition of the local skiing frame coordinates. The *x*-axis is parallel to the CoM velocity vector, the z-axis is the cross-product of the *x*-axis and the gravity (i.e., vertical axis), and the y-axis is the cross product of the *z*- and *x*-axes. The dotted line indicates the trajectory of the athlete’s CoM.

For each run the 3D trajectory error *d*(*t*) was obtained with *d*(*t*) = *p*_CoM, inertial_(*t*) − *p*_CoM,ref_(*t*). The norm of the trajectory difference, i.e., *d*_tot_(*t*) = ||*d*(*t*)||, was used to evaluate the error with respect to the reference system. To allow a better error description, *d*(*t*) was also expressed in the local skiing frame 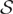 [^
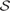
^*d*(*t*)] which was defined as follows: the *x*-axis was pointing along the reference CoM velocity vector, the *z*-axis was the cross-product of the *x*-axis and the gravity vector, and the *y*-axis was the cross product of the *z*- and *x*-axes (**Figure 4**). Next, per run-accuracy and precision were calculated with the average and standard deviation of *d*_tot_(*t*) and ^
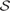
^d(t), respectively. Overall accuracy was then defined as the average of all per-run accuracies and overall precision was defined as the average of all per-run precisions. The total speed error *S*_tot_(*t*) was defined as the difference of the speed norms: *S*_tot_(*t*) = ||*v*_CoM,inertial_(*t*)|| − ||*v*_CoM,ref_(*t*)||. ^
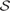
^*S*(*t*) was obtained the same way as ^
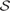
^*d*(*t*).

#### Performance Parameters Derived From CoM Kinematics

**FIGURE 5:**
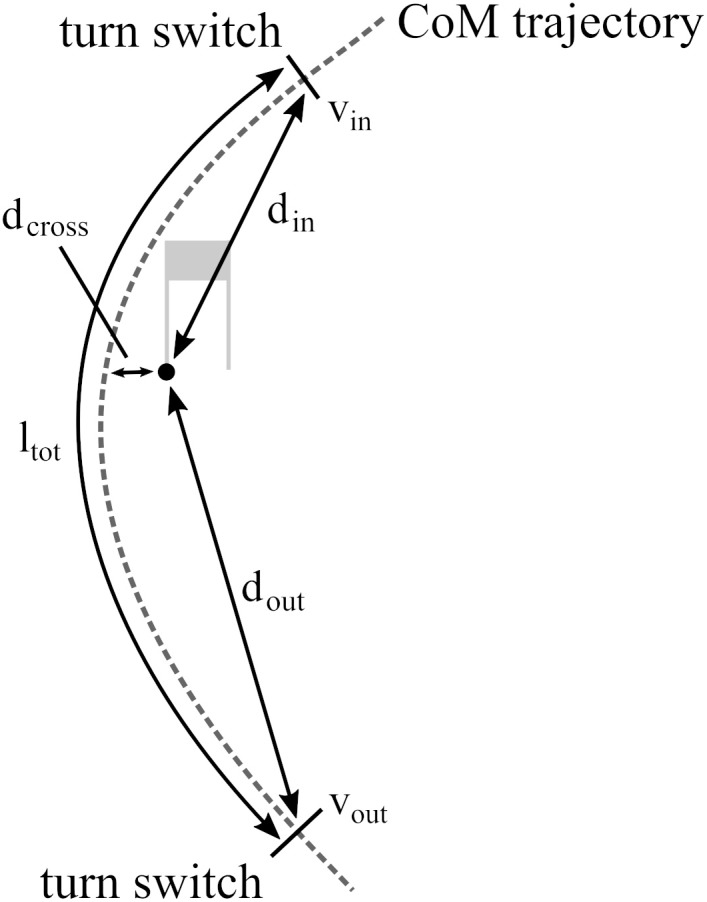
Illustration of a turn’s distance and speed performance parameters. A turn starts and stops at a turn switch and speed and position at these moments as well as at gate grossing are then expressed in relation to the gate position.

In order to validate whether the proposed system was sensitive enough to detect changes in performance, for one representative turn, five performance parameters were computed with both the reference and the inertial sensor-based system and for all runs. In analogy to a previous study by (Spörri et al., 2012) the performance parameters compared were: *d*_in_ distance from turn switch marking the beginning of the turn to the gate position, *d*_out_ distance from turn switch marking the end of the turn to the gate position (**Figure 5**). For these two events the instantaneous CoM speed norm (*v*_in_, *v*_out_) were extracted. For the same turn, total 3D CoM trajectory length *l*_tot_ was computed. In addition, the CoM distance to the gate at gate crossing (*d*_cross_) was extracted to evaluate the relative anchor point estimation. The beginning of a turn (i.e., turn switch) was detected based on the criterion of equal left/right ankle distance to the athlete’s CoM (Fasel et al., 2016b). The parameter results were then compared based on Bland–Altman plots and LoAs were computed (Bland and Altman, 1999).

## Results

### CoM Kinematics

The trajectory’s overall accuracy and precision were 0.24 and 0.09 m for position, and 0.00 and 0.18 m/s for speed (**Table 1**). Errors were similar along each axis in the local skiing frame *S*. On average, one to two gates per run could not be detected by the magnetometers because the athlete passed too far from a gate; usually the first and/or last gate were not detected. It was observed that the magnetic field created by the magnets could always be detected up to a distance of approximately 0.80 m. Increasing the distance between available anchor points for trajectory drift correction decreased the accuracy and precision (**Table 1**).

**Table 1:** Average (standard deviation) accuracy and precision for the total error and the error along each local skiing axis for speed and position.

		All gates		Simulated DH	
		Accuracy	Precision	Accuracy	Precision
**Speed, m/s**	Total error	0.00 (0.02)	0.18 (0.02)	0.01 (0.03)	0.31 (0.14)
	*X*-Axis	−0.01 (0.01)	0.30 (0.04)	0.00 (0.01)	0.33 (0.05)
	*Y*-Axis	0.00 (0.01)	0.20 (0.03)	−0.01 (0.03)	0.33 (0.14)
	*Z*-Axis	0.00 (0.00)	0.21 (0.08)	0.00 (0.01)	0.22 (0.08)
**Position, m**	Total error	0.24 (0.09)	0.09 (0.03)	0.34 (0.10)	0.19 (0.14)
	*X*-Axis	0.01 (0.10)	0.14 (0.03)	0.00 (0.12)	0.18 (0.04)
	*Y*-Axis	0.02 (0.13)	0.10 (0.02)	0.03 (0.13)	0.25 (0.18)
	*Z*-Axis	0.01 (0.10)	0.07 (0.04)	0.01 (0.11)	0.08 (0.04)

*All values were obtained with the inertial sensor-based system only with surveyed anchor points. For the simulated downhill (DH) only every third anchor point was used for the fusion.*

### Performance Parameter-Related Findings

**FIGURE 6:**
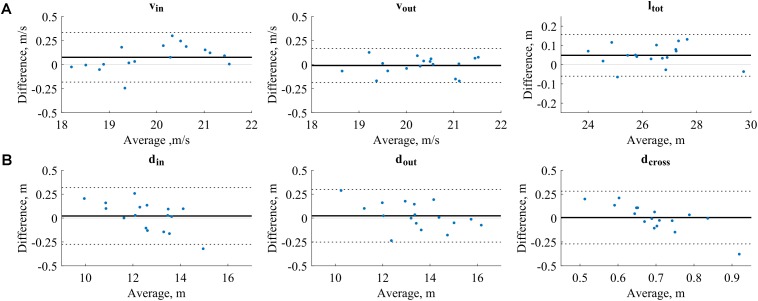
Bland-Altman plots for the performance parameter validation. The solid black lines mark the mean error and the dashed black lines the limits of agreements.

Limits of agreement were between −0.27 and 0.32 m for position, between −0.19 and 0.33 m/s for speed, and −0.06 and 0.16 m for path length (**Table 2**). With the exception of gate distance at gate crossing, LoAs were up to five times smaller than the performance parameter’s standard deviation (**Table 2**). Gate distance error seemed to depend on the distance: small gate distances were overestimated and large gate distances were underestimated (**Figure 6**).

**Table 2:** Average parameter values and error mean with LoA for the extracted performance parameters.

	Parameter value		Error		
	Average	Std	Lower LoA	Mean	Upper LoA
*v*_in_, m/s	19.94	1.04	−0.18	0.08	0.33
*v*_out_, m/s	20.30	0.82	−0.19	−0.01	0.17
*d*_in_, m	12.59	1.29	−0.27	0.02	0.32
*d*_out_, m	13.41	1.56	−0.25	0.02	0.30
*d*_cross_, m	0.70	0.10	−0.27	0.01	0.28
*l*_tot_, m	26.35	1.38	−0.06	0.05	0.16

## Discussion

In this study, alpine ski racing gates were equipped with magnets, and their positions were fused with magnetic and inertial sensor measurements to obtain drift-free absolute 3D CoM kinematics (trajectory and speed) of the skier. Gate and magnet positions (i.e., anchor points) were determined using surveying technology. Considering that the sacrum would not pass the anchor points with zero distance, the position difference between the athlete’s sacrum and anchor points was estimated based on the athlete’s posture and the peak magnitude of the magnetic field. Absolute CoM kinematics were obtained by adding the estimated CoM relative to the LJC to the estimated absolute LJC trajectory. The measurement performances of the system to estimate CoM trajectory and speed as well as ski performance parameters were estimated against a differential GNSS as reference with 17 runs on a GS course and a simulated DH.

### Accuracy and Precision of CoM Kinematics

We found good accuracy and precision for both CoM position (0.24 and 0.09 m) and speed (0.00 and 0.18 m/s) for GS (**Table 1**). In the context of alpine ski racing, no other study proposed to compute 3D CoM kinematics based on inertial sensors and surveyed anchor points. (Brodie et al., 2008) used a low-cost global positioning system (GPS) sampling at 1 Hz and fused position and speed data with acceleration obtained from inertial sensors. In addition, the start and finish points were used as anchor points for removing position offsets of the GPS. Nevertheless, over a 300 m run errors of up to ±1.5 m were reported. For differential GNSS (Gilgien et al., 2014) reported antenna position error standard deviations of <0.05 m. Using the same system but for CoM trajectories, (Gilgien et al., 2015c) reported error standard deviations of 0.12 m for position and 0.19 m/s for speed. Thus, even though the proposed system did not use differential GNSS the observed errors were comparable to the above systems.

When removing anchor points to simulate a DH race, position accuracy and precision decreased from 0.24 and 0.09 m to 0.34 and 0.19 m, respectively, as expected (**Table 1**). Instead of a position update in the EKS filter approximately every 1.5 s, such an update could only be performed approximately every 4.5 s. Interestingly, errors in the horizontal plane increased more than along the vertical axis (**Table 1**). This could most likely be due to the law of the cosine for the removal of the gravity on the vertical axis and the horizontal plane. Since 1 - cos(ε) << sin(ε), for a small inclination error ε due to drift, the partly erroneous gravity removal has only little effect on the vertical axis compared to the horizontal plane. Thus, much less error could accumulate along the vertical axis compared to the horizontal plane.

**FIGURE 7:**
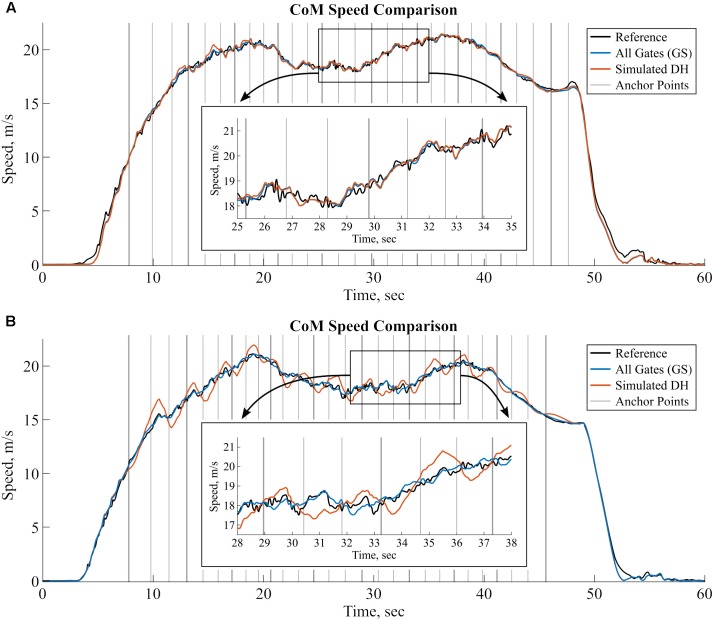
CoM Speed comparison for two typical runs with different error behavior for the simulated DH. In both plots, reference CoM speed is shown in black, inertial sensor-based CoM speed with all anchor points (GS) is shown in blue, and the simulated DH is shown in red. Anchor points are marked with the vertical gray lines. For GS all anchor points were considered whereas for simulated DH only the anchor points marked in bold were considered. **(A)** A run with little to no change of speed between GS and simulated DH. **(B)** Another run with large precision decrease for simulated DH compared to GS.

We could also observe two different error behaviors when switching from GS to simulated DH: for part of the measurements the precision did not decrease much (**Figure 7A** shows an example run) while for the other part of the measurements the precision did decrease much more (**Figure 7B**). Depending on the turn direction, the CoM speed for simulated DH was either over- or under-estimated and was close to the reference value at each gate crossing, even at the ones were no anchor point was available. This suggests that for some of the runs, a movement-dependent speed bias was present that the EKS was not able to completely remove. One cause could be an insufficient modeling of the EKS which was designed and optimized for one turn between each anchor point whereas the simulated DH had three turns between each anchor point. For our simulated DH condition, a different design of the EKS should maybe be used to correct these errors. Nevertheless, for real DH with one turn between each anchor point, we expect that the proposed EKS would lead to a better precision than found with the simulated EKS in this study. A new study with real DH conditions should be performed in order to confirm the results of the simulated DH.

### Limits of Agreement for CoM-Derived Performance Parameters

Spörri et al. (2012) observed turn entrance and exit speed and distance differences of at least 0.3 m/s and 0.3 m, respectively, for comparisons between the fastest and slowest runs of the same athlete in GS. The LoA found in this study are of the same magnitude (**Table 2**). However, for total turn COM trajectory length, LoA were below the reported difference of 0.3 m between the fastest and slowest trial reported in (Spörri et al., 2012) for GS. Therefore, the system’s resolution might be at the limit for detecting instantaneous performance-related differences such as speed and position at a certain point but may be well suited for “averaged” performance-related differences such as trajectory lengths in GS. To assess speed differences between athletes (Gilgien et al., 2016a) or speed differences caused by different types of skis in the same athlete (Gilgien et al., 2016b) in DH, the conclusion with respect to accuracy of the proposed system is similar as for GS. Differences between single runs might be hard to detect due to the fact, that the accuracy for speed of the proposed system is about equal to the differences expected between athletes or ski interventions.

### Limitations

A first limitation of the study was the constraint that the athletes had to pass each anchor point sufficiently close so that the perturbation in the Earth magnetic field caused by the buried magnet could be detected reliably. The magnets used in this study allowed detecting gate crossings up to distances of approximately 0.80 m. For the technical disciplines of slalom and GS and elite athletes this is no problem: they pass most of the gates as close as possible. Therefore, their sacrum passes the gate rarely with a distance larger than this limiting distance. However, with lower level athletes and in the speed disciplines super-G and DH gates may be passed with larger distances. This could be counter-acted by increasing the strength of the magnets or by placing several magnets along a line perpendicular to the expected ski trajectories and, in consequence, an adapted EKS.

A second limitation of the study was that the gates still had to be surveyed using a differential GNSS or a tachymeter. Thus, even though the athletes do not need to wear an expensive and sometimes difficult to handle differential GNSS, such a system was still needed for the gate surveying. For certain applications where relative position and speed information is sufficient, it might be possible to average anchor point positions computed from all runs on the same track (Fasel et al., 2017a) and leave out the surveying work. Another possibility would be to use a similar approach but including a low-cost GNSS worn by the athletes (Fasel et al., 2017b).

Despite the fact that the fusion of anchor points with inertial sensors allowed correcting speed and position drift, such performance would probably not have been possible without a considerable pre-processing effort. The sensors’ offsets and sensitivities were carefully calibrated prior to the measurements. Moreover, sensor orientation drift was reduced prior to the EKS with the joint drift reduction procedure (Fasel et al., 2017d, 2018). This allowed estimating sensor orientations with dedicated, non-linear and precise methods, instead of directly including orientation estimation and drift reduction by means of a general model in the EKS. Thus, the EKS could be kept as simple as possible (i.e., with a minimum number of states and only few filter parameters needed to be tuned). The employed EKS was considered as *a means to an end* instead of forming the core of the study. The filter parameters were only chosen empirically and more work should be spent on properly tuning these parameters in a future study. The system’s performance could also be improved by a better estimation of the relative position of the anchor points with respect to the sacrum. The estimation of the total distance between the sacrum and the anchor point (i.e., magnet) based on the measured magnetic peak intensity could involve some errors: it was highly probable that the magnetic peak field intensity was underestimated because of the magnetometer’s low sampling rate of 125 Hz. At 20 m/s (i.e., 72 km/h) the athlete covers 0.16 m per sample. Therefore, it is likely that the magnetic intensity was not sampled exactly at its peak. Peak intensity could be measured more reliably by increasing the sampling rate and designing an advanced curve fitting and peak identification algorithm. Moreover, the magnetic field intensity created by the magnet decreases with the third power of the distance. Therefore, small measurement errors for low intensities can lead to large errors for the distance estimation. Stronger magnets would increase the generated magnetic field and lead to a more reliable distance estimation. At the same time, fewer gates would be missed since the magnetic disturbance could also be measured for gate distances larger than 0.80 m.

## Conclusion

The proposed system that fuses inertial sensors with periodically available anchor point positions allowed obtaining CoM kinematics with a higher accuracy and precision than with a system solely based on a low-cost GNSS (Brodie et al., 2008; Gilgien et al., 2014). Moreover, the proposed system’s performance was close to that of geodetic differential GNSS (i.e., reference system). The independency of the proposed system from the use of GNSS allows its application also in indoor situations, such as in skiing halls.

## Author Contributions

BF, MG, and JS conducted the data collection. BF, MG, JS, and KA conceptualized the study design, contributed to the analysis and interpretation of the data. BF drafted the manuscript, all other authors revised it critically. All authors approved the final version and agreed to be accountable for all aspects of this work.

## Conflict of Interest Statement

The authors declare that the research was conducted in the absence of any commercial or financial relationships that could be construed as a potential conflict of interest.
